# Complicated lower rectal perforation secondary to ingested meat bone foreign body injury: a case report

**DOI:** 10.1093/jscr/rjag687

**Published:** 2026-08-08

**Authors:** Mohamed H Ahmed, Mohamed H Fadul, Moayad Elgassim, Albarra Mubasher, Alharith Alhussien, Ejaz Ahmed Latif, Husham M A Abdelrahman

**Affiliations:** Medical Education Department, Hamad Medical Corporation, Doha, Qatar; Medical Education Department, Hamad Medical Corporation, Doha, Qatar; Medical Education Department, Hamad Medical Corporation, Doha, Qatar; Medical Education Department, Hamad Medical Corporation, Doha, Qatar; Faculty of Medicine, University of Gezira, Wad Medani, Sudan; Colorectal Surgery Unit, General Surgery Department, Hamad Medical Corporation, Doha, Qatar; Hamad Trauma Surgery, Department of Surgery, Hamad Medical Corporation, Doha, Qatar

**Keywords:** perianal abscess, rectal perforation, foreign body, bone ingestion, anal pain

## Abstract

Most ingested foreign bodies (FBs) commonly pass through the gastrointestinal tract without complications. However, sharp objects, such as bones, may cause mucosal injury, perforation, and abscess formation. Rectal impaction is rare, but can pose a diagnostic challenge, especially in nonspecific presentations, such as anal pain. We report a case of a 40-year-old male patient who first presented with severe acute anal pain. He was later found to have complex anorectal abscesses and a rectal perforation secondary to the manual removal of an ingested meat bone FB. Surgical exploration revealed a full-thickness rectal perforation and anorectal abscesses, which were subsequently drained, and the perforation was repaired. This case highlights the importance of considering FB related complications in patients presenting with anal pain, especially following manipulation. Prompt imaging and surgical intervention are key to preventing severe outcomes.

## Introduction

Acute anal pain is a frequent anorectal symptom of diverse etiologies, including anal fissures, thrombosed hemorrhoids, and abscesses [1]. Proper history taking, rectal examination, in addition to clinical imaging, play an important role in the workup. Anorectal abscesses are infectious and suppurative conditions that commonly develop due to the infection of anal glands in the intersphincteric plane. Obstruction of anal crypts is a common etiology of anorectal abscesses [2].

Foreign body (FB) ingestion is a common complaint in the gastrointestinal tract that presents in the emergency department (ED). It may occur in both the adult and pediatric populations. Most ingested FBs pass spontaneously without intervention (80%–90%) [3]. Perianal abscess due to an ingested FB is rare [4].

Several factors may determine the complications and severity, including the type of ingested object and its physical properties, the site of impaction, and the time since ingestion [4]. The most common sites of impaction and perforation include the appendix, cecum, and the terminal ileum. The anal canal is an unusual site of FB impaction. Anal stenosis or spastic anal sphincter may predispose to FB impaction in the perianal canal [1].

Bones are often sharp objects that can get lodged in the digestive tract and cause perforation and abscess formation. While cases of chicken and fish bone ingestion have been previously described, we report an exceptional case of rectal perforation and perianal abscesses secondary to ingested meat bone FB injury.

Ethical approval was obtained from the Medical Research Council at Hamad Medical Corporation, the local Institutional Review Board (IRB). This manuscript was prepared using applicable EQUATOR CARE guidelines for case reports. The CARE Checklist has been completed by the authors for this case report, attached as online supplementary material.

## Case presentation

A 40-year-old male with a past medical history of hypertension, and irritable bowel syndrome, presented initially to the ED with a 4-day history of sudden-onset severe anal pain, mild abdominal pain, and constipation. On examination, his abdomen was soft, lax, and mildly distended. Per rectal examination revealed no fissures or hemorrhoids. Abdominal X-ray showed no air-fluid levels or bowel dilation and ruled out intestinal obstruction. His blood tests were within the normal ranges except for a white cell count (WCC) of 14.9 × 10^3^/μl. The patient was able to pass stools following the administration of rectal enema and analgesics and was subsequently discharged home.

Three days later, he re-presented again to the ED with fever, severe anal pain, lower abdominal pain, vomiting, dysuria, and constipation. This time, he disclosed manually removing a 5–6 cm piece of meat bone from his anal canal during defecation three days prior. On examination, his abdomen was soft, lax, and non-tender. Inspection of the perianal area was unremarkable. A digital rectal exam was notable for tenderness, particularly at the 7–12 o'clock position, which was aggravated by coughing. No site of induration or fluctuation could be appreciated. Proctoscopy was intolerable. He was admitted and started on IV piperacillin/tazobactam and supportive management. Blood tests showed a WCC of 20.9 × 10^3^/μl and a C-reactive protein (CRP) level of 198.9 mg/L. Septic workup, including blood cultures, was negative.

**Figure 1:**
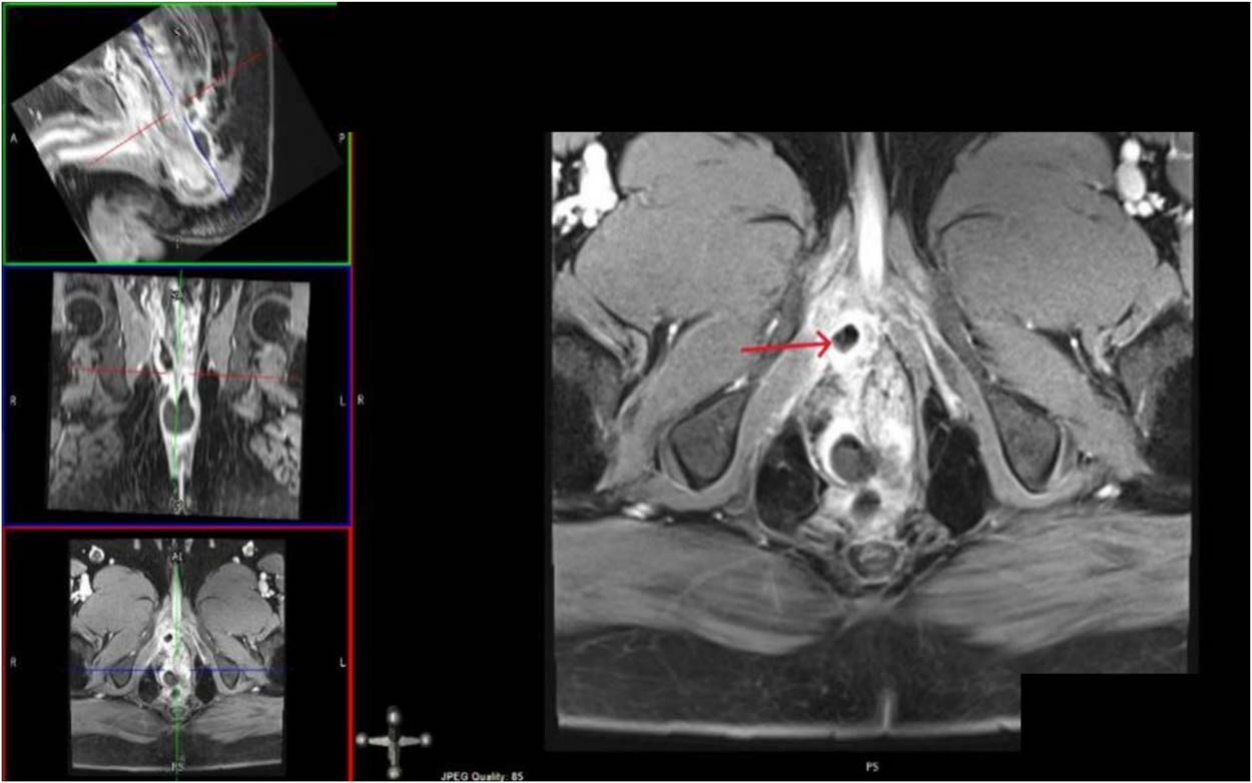
MRI of the anus revealing a small anterior abscess (arrow).

**Figure 2:**
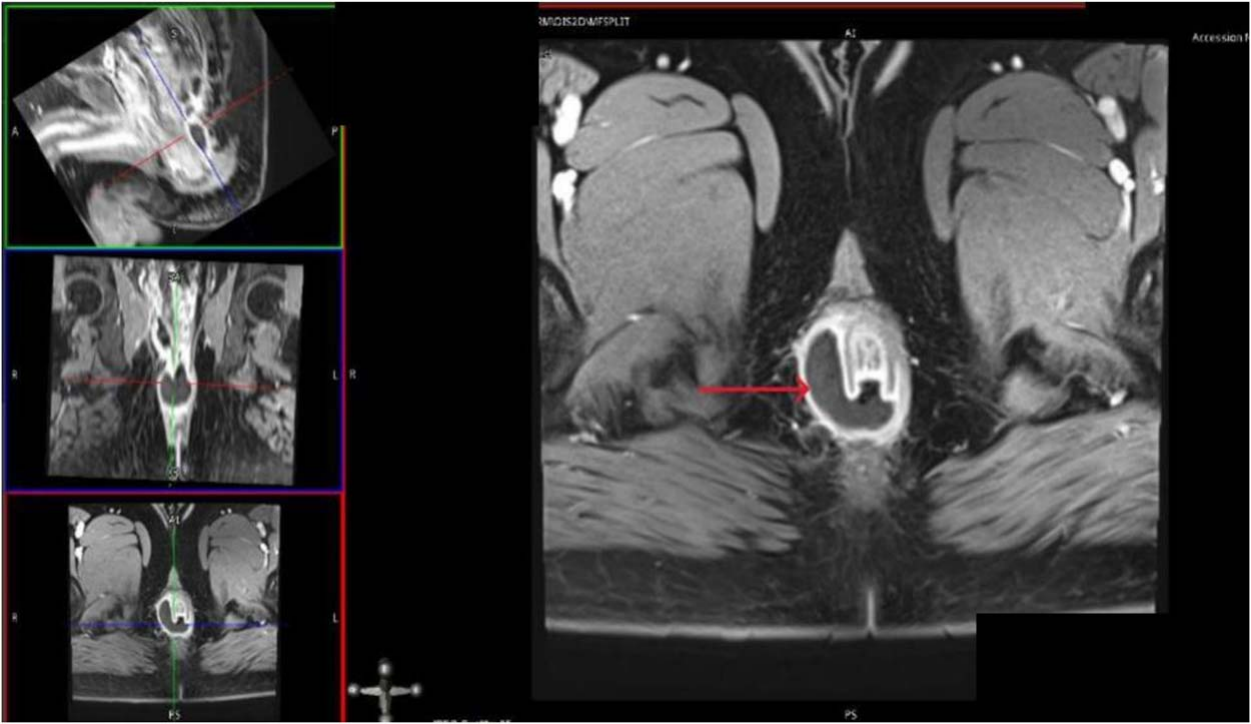
MRI of the anus revealing right inferior abscess (arrow).

**Figure 3:**
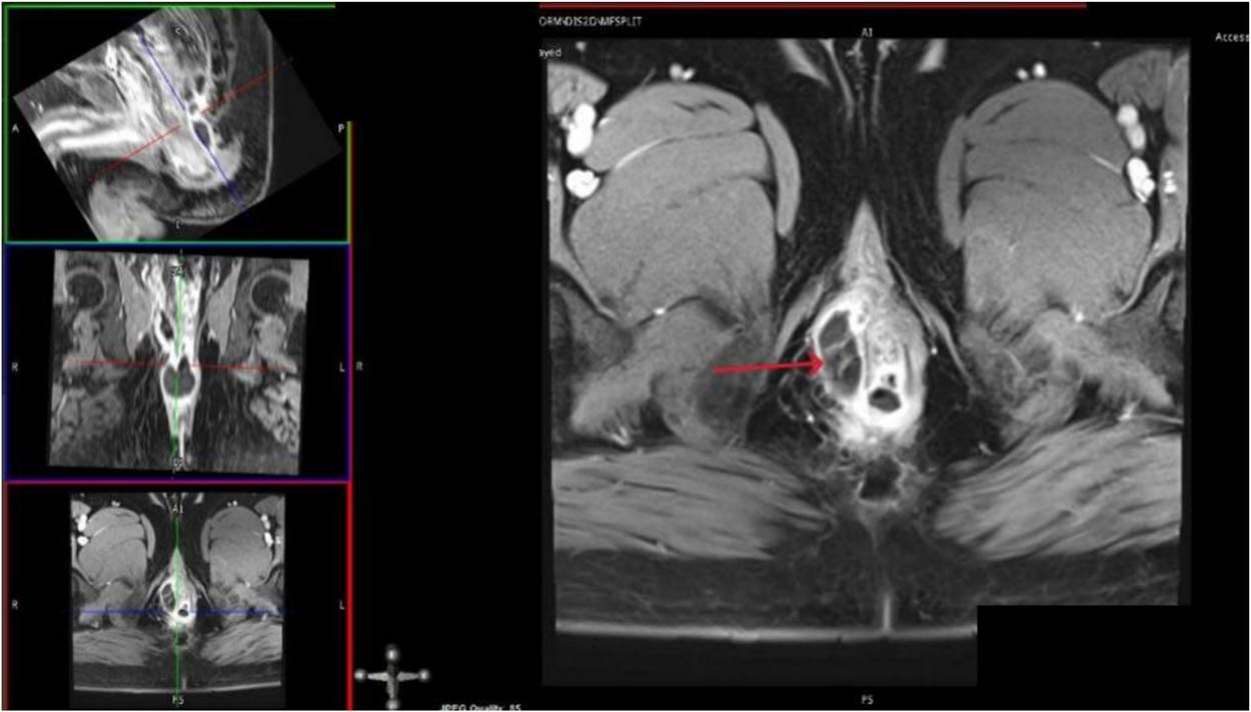
MRI of the anus revealing the septae within the abscess.

Magnetic resonance imaging (MRI) of the pelvis with IV contrast (Fig. 1) showed diffuse oedema and inflammatory changes of the anal canal and rectum, along with loculated interconnected right and posterior perianal abscesses with an internal opening at the 6 o'clock position about 4 cm above the anal verge (Fig. 2). The largest pocket measures 7 × 3 cm. The abscesses were stretching out the right ischiorectal fossa, pushing the anal canal to the left side, reaching and abutting the levator ani and obturator internus muscles (both demonstrating inflammatory changes), and anteriorly reaching the penis bulb and prostate apex on the right side with no direct involvement. Another small abscess, measuring 1 × 1.5 cm, with an internal opening at 12 o'clock position just at the level of the anal verge was noted (Fig. 3). It was also notable for significant mesorectal and pelvic inflammatory changes with a trace of fluid in the pelvis, multiple reactive mesorectal lymph nodes (up to 4 mm) and right iliac lymph nodes (up to 6 mm), and subcutaneous oedema of the perineum.

The colorectal surgery team was consulted, and the patient was posted for a perianal exam under anesthesia (EUA) with an incision and drainage (I&D) procedure. However, during the procedure, a 2 cm rectal perforation was identified at the 6 o'clock position, 2 cm from the anal verge, connecting with a pus-filled intersphincteric cavity. The cavity was incised and drained, and the rectal perforation was repaired using a polydioxanone suture, and a Penrose drain was inserted. Postoperatively, the patient was vitally stable, and the wound was clean and dry. He was discharged on cefuroxime, metronidazole, laxatives, and analgesics. When reviewed in the outpatient clinic a few days later, he complained of anal pain. The wound was cleaned and dressed, and the drain was removed.

Two weeks after his initial presentation, he again presented to the ED with fever and perianal pain, swelling, and purulent discharge. PR examination was remarkable for a new, tender, fluctuating mass. His WCC was 12.6 × 10^3^/μl, and CRP level was 19.7 mg/L. IV ciprofloxacin and metronidazole were started. He was also taken to the operating theater for an EUA, where an intersphincteric perianal abscess, fistulizing with rectum at 12 o'clock position, was incised, drained, washed with hydrogen peroxide and saline, packed with Vaseline gauze, and dressed. The area of the previous rectal repair was palpable with a defect at 6 o'clock and a clean incision site with no discharge. Notably, the previous MRI had revealed a small abscess with an internal opening at 12 o'clock, as described above. Postoperatively, the patient was vitally stable, and his pain had improved. He was then discharged home with a follow-up appointment after 1 week, where he was afebrile and pain-free, and the wounds were clean and non-tender.

## Discussion

Ingestion of FBs has been described as a common event, especially in populations, such as alcoholics, the mentally impaired, children, and those who eat hastily. Most ingested FBs are of organic dietary origin, such as chicken and fish bones [5]. The majority—approximately 80%—pass through the gastrointestinal tract without causing injury or complications [5]. However, in the remaining 20% of cases, FBs may become impacted, carrying the potential for serious complications, including obstruction, perforation, fistulation, abscess formation, and hemorrhage [6]. Thorough history-taking plays a crucial role in identifying the ingestion incident, its timing, the properties of the FB, and its passage through the GI tract, thereby guiding both the investigative process and the management plan.

FB impaction typically occurs at anatomical sites predisposed to physiological narrowing, angulation, or structural abnormality, including the upper and lower esophageal sphincters, pylorus, duodenum, ileocecal valve, caecum, appendix, sigmoid colon, and anus. Sites of prior adhesions, surgical anastomosis, and diverticula may also retain FBs. Gastrointestinal perforation secondary to FB ingestion remains rare, reported in approximately 1% of cases, with the most common perforation sites being the ileocecal valve and rectosigmoid junction—areas characterized by acute angulation and transitional anatomy [5, 6]. The risk of perforation is principally determined by the physical and morphological properties of the ingested object, notably its length and sharpness [6].

Anorectal abscesses arise from infection of the anal glands, which are located within the intersphincteric space and drain into the anal crypts at the dentate line. Obstruction of these glands leads to stasis, bacterial overgrowth, and suppuration. The resulting infection spreads along the path of least resistance through the intersphincteric plane, giving rise to the classic anorectal abscesses [7].

In the present case, we speculate that the anorectal abscess was not a direct consequence of FB migration through the bowel wall, but rather the result of trauma sustained during its manual evacuation. The forcible removal of a 5–6 cm meat bone per rectum likely caused mucosal injuries at both the 6 o'clock and 12 o'clock positions, with the injury at 6 o'clock being more extensive and ultimately associated with a full-thickness rectal perforation identified intraoperatively. These mucosal breaches likely served as entry points for secondary infection, leading to the intersphincteric and ischiorectal abscesses documented on imaging. The initial MRI had already demonstrated a separate small abscess with an internal opening at the 12 o'clock position, further supporting the hypothesis of a dual-site traumatic insult sustained during the same manipulative episode.

Clinicians should note that a deep-seated anorectal abscess may be present even when external perianal examination appears unremarkable, as illustrated by this case. On the patient’s second presentation, inspection of the perianal area was entirely unremarkable despite the presence of large, complex, multiloculated abscesses extending into the ischiorectal fossa. This presentation can be explained by that deep abscesses track along fascial planes and may remain entirely below the skin surface, producing no visible signs of inflammation, consistent with previously reported case of anorectal abscess [7]. In this case, the only external clue was localized tenderness on digital rectal examination at the 7–12 o'clock position, aggravated by coughing. The markedly elevated inflammatory markers, disproportionate to the benign external appearance, highlight the severity of this condition.

Prompt and appropriate imaging is essential in such cases. While CT is often the first-line modality in acute settings given its speed and availability, MRI is the preferred investigation for complex jh sepsis due to its superior soft tissue resolution and ability to delineate fistulous tracts and abscess extensions—factors with direct implications for surgical planning and fistula recurrence rates [7]. The correlation between the MRI-identified internal openings at both 6 and 12 o'clock positions and the intraoperative findings in our patient exemplifies the diagnostic precision MRI holds and reinforces its central role in guiding management in cases of this complexity.

Management of FB-associated anorectal complications is guided by the patient’s clinical status, the morphological characteristics of the FB, and the extent of sepsis. Broad-spectrum antibiotics and surgical drainage of the septic focus remain the cornerstones of treatment, with open I&D being the established standard of care despite reports of minimally invasive alternatives in the literature [6, 7]. In our patient, management adhered to these principles, and the identification of the rectal perforation intraoperatively necessitated primary repair—an additional step that highlights the value of thorough surgical exploration under anesthesia rather than reliance on imaging alone.

## Conclusion

This case illustrates a rare but serious complication of manual FB extraction complicated by rectal perforation and complex multiloculated anorectal abscess. Three learning points emerge. First, manual extraction of an impacted FB is an under recognized cause of anorectal abscess, distinct from direct FB migration. Second, a normal perianal inspection does not exclude deep-seated abscess. Third, early MRI is critical to delineating disease extent and internal openings, directly informing surgical strategy. EUA with I&D, supported by broad-spectrum antibiotics, remains the definitive intervention.

## Data Availability

All data generated or analyzed during this study are included in this article. Further enquiries can be directed to the corresponding author.

## Study approval statement

This study was reviewed and approved by the Medical Research Center at Hamad Medical Corporation, approval number [MRC-04-25-1278].
